# Publisher Correction: Tracing the transitions from pluripotency to germ cell fate with CRISPR screening

**DOI:** 10.1038/s41467-018-07765-y

**Published:** 2018-12-11

**Authors:** Jamie A. Hackett, Yun Huang, Ufuk Günesdogan, Kristjan A. Gretarsson, Toshihiro Kobayashi, M. Azim Surani

**Affiliations:** 1 Wellcome Trust/Cancer Research UK Gurdon Institute, University of Cambridge, Tennis Court Road, Cambridge CB2 1QN, UK; 2 Epigenetics and Neurobiology Unit, European Molecular Biology Laboratory (EMBL), via Ramarini 32, 00015 Rome, Italy; 3 Department of Physiology, Development and Neuroscience, University of Cambridge, Downing Street, Cambridge CB2 3DY, UK; 4 Department of Developmental Biology, University of Göttingen, Göttingen Center for Molecular Biosciences, Justus-von-Liebig Weg 11, 37077 Göttingen, Germany; 5 Present Address: Center for Genetic Analysis of Behaviour, National Institute for Physiological Sciences, 5-1 Higashiyama Myodaiji, 444-8787 Okazaki, Aichi Japan

Correction to: *Nature Communications*; 10.1038/s41467-018-06230-0; published online 16 October 2018

The original version of this Article contained errors in the author affiliations.

Ufuk Günesdogan was incorrectly associated with Center for Genetic Analysis of Behaviour, National Institute for Physiological Sciences, 5-1 Higashiyama Myodaiji, Okazaki, Aichi 444-8787, Japan and Toshihiro Kobayashi was incorrectly associated with Department of Developmental Biology, University of Göttingen, Göttingen Center for Molecular Biosciences, Justus-von-Liebig Weg 11, 37077 Göttingen, Germany.

This has now been corrected in both the PDF and HTML versions of the Article.

