# Supplementary material for: Tracing the transitions from pluripotency to germ cell fate with CRISPR screening
Source: Nat Commun. 2018 Oct 16;9:4292. doi: 10.1038/s41467-018-06230-0 (PMC6191455; doi:10.1038/s41467-018-06230-0)
Supplement: Supplementary file 1 — Supplementary Information [file 41467_2018_6230_MOESM1_ESM.pdf]

Tracing the Transitions from Pluripotency to Germ Cell Fate with CRISPR Screening

Hackett et al

Supplementary Information

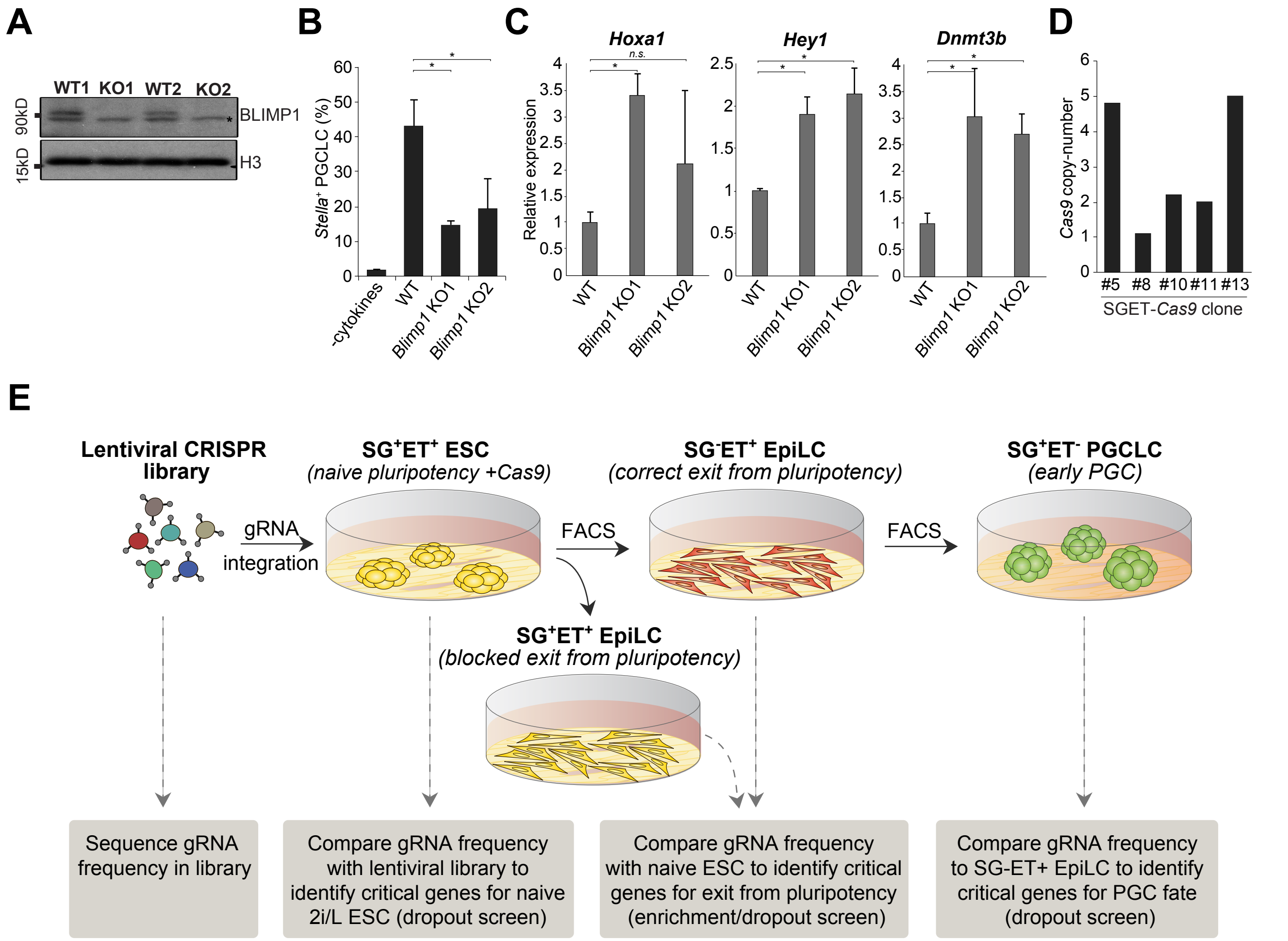

Supplementary Figure 1. Screen design and validation of the SGET reporter. (A) Western blot for BLIMP1 using *Blimp1* KO (KO1, KO2) and wild type (WT1, WT2) SGET cell lines, * indicates non-specific band. (B) FACS analysis at day 5 shows the efficiency of PGCLC specification is greatly impaired in *Blimp1-/-* SGET lines relative to WT SGET control. The degree of impairment (2-3 fold) is comparable to the reduction of migratory PGC numbers in *Blimp1-/-* embryos *in vivo,* implying SGET PGCLC recapitulate *in vivo* phenotype. -cytokines performed with wild type. (C) qRT-PCR gene expression analysis of remaining PGCLC generated from *Blimp1-/-* SGET lines, showing aberrant activation of somatic genes relative to WT, similarly to *in vivo Blimp1* mutant PGC. Each independent line was assayed in biological duplicate using >12 independent inductions, with significance determined using a one-tailed T-test; * indicates $p<0.05$. (D) Copy-number of *Cas9* integrated into the genome of SGET clones by PiggyBac determined with qRT-PCR, relative to a single-copy (diploid) gene on Chr17. Clone #8 and #10 were selected for further analysis. (E) A lentiviral library of >87,000 gRNAs is integrated into the genome of SGET ESC (expressing *Cas9*) at low MOI to ensure high probability of a single gRNA integration per cell. The normalised frequency of each gRNA in the lentiviral and ESC population is determined by amplifying all gRNA sequences from genomic DNA of the population and deep sequencing. The loss/depletion of multiple gRNAs targeting the same gene in the ESC population, relative to the starting lentiviral library, indicates the targeted gene is essential/important for naive ESC (dropout screen).This principle of comparing gRNA frequency to preceding populations is then applied during cell fate transitions toward PGCLC. Importantly, the SGET reporter is used to iteratively purify cells via cytofluorometry that have appropriately transitioned to each stage thereby ensuring indirect effects of knockouts that influence earlier stages/cell identities are excluded. Thus, in each transition only gRNAs targeting genes required for that specific cell fate decision become depleted, since all general cell survival genes/gRNAs and gRNAs for preceding states are already lost. This enables critical genes for each transition to be identified.

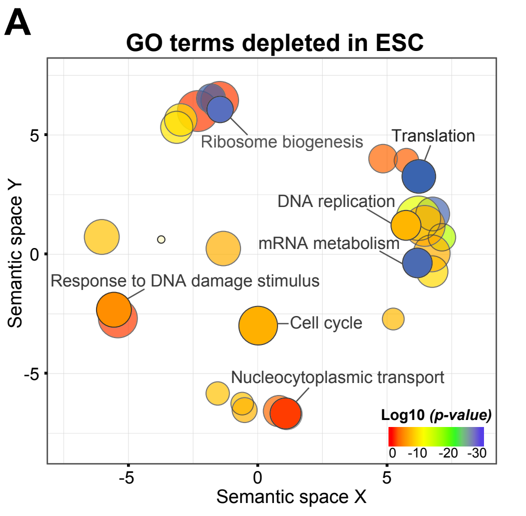

Top 5 KEGG pathway	*p-value* (Bonferroni)
Proteasome	$1.7e^{-17}$
DNA replication	$2.8e^{-11}$
Spliceosome	$3.3e^{-11}$
Ribosome	$2.4e^{-10}$
Aminoacyl-tRNA biosynthesis	$1.1e^{-08}$

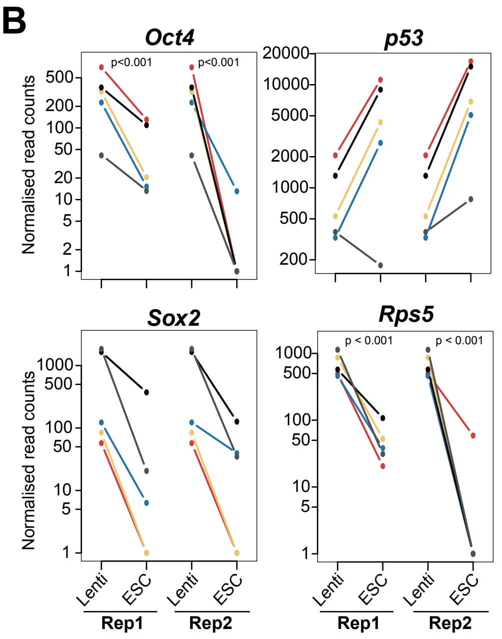

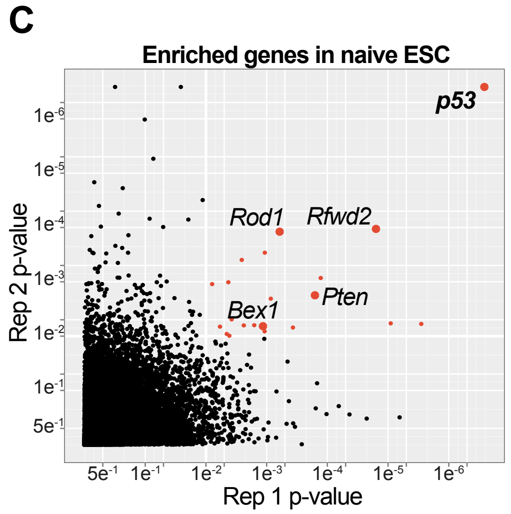

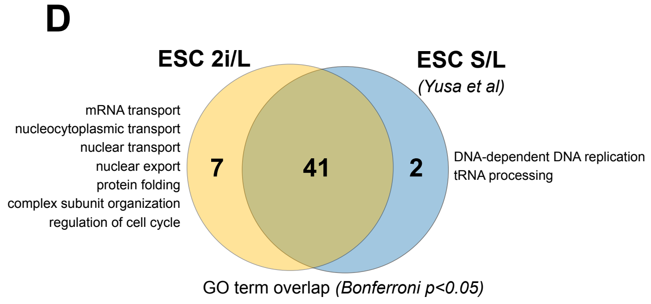

Cell type	Number of cells sorted	
	r1	*r2*
ESC	10m	10m
SG⁻ET⁺ EpiLC	9.8m	12.2m
SG⁺ET⁺ EpiLC	1.6m	2.1m
SG⁺ET^{low} PGCLC	1.2m	1.7m
Soma	2.3m	9.2m

Supplementary Figure 2. CRISPR screen analysis in naive ESC. (A) Representation of significant gene ontology (GO) terms linked with depleted gRNAs (corresponding to essential genes) in naive ESC after lentiviral library integration. The most significant terms correspond to broadly critical biological processes such as ribosome biogenesis and translation, suggesting the library is effectively disrupting targeted gene function. Significant KEGG pathways among depleted genes are shown below. Depletion of these general cellular survival genes in ESC facilitates screening of critical genes specifically for subsequent cell fate decisions without confounding effects of such essential genes, since the ESC are already normalised for their absence. (B) Plot of the normalised frequency of selected gene targeting gRNAs in the initial lentiviral library and in ESC across replicates. Each colour represents a unique gRNA. Critical pluripotency genes such as *Oct4,* or general survival genes such as *Rps5,* become depleted by independent gRNAs, whereas *p53* is enriched. (C) Scatter plot showing significantly enriched genes in ESC after knockout (ESC carrying gRNA/gene knockout harbour a selective/proliferative advantage). (D) Venn diagram showing a comparison of significant gene ontology categories among depleted (essential) genes for ESC in 2i/L and ESC in Serum/Lif (S/L). Below is a table detailing the number of sorted cell for each replicate of the CRISPR screen.

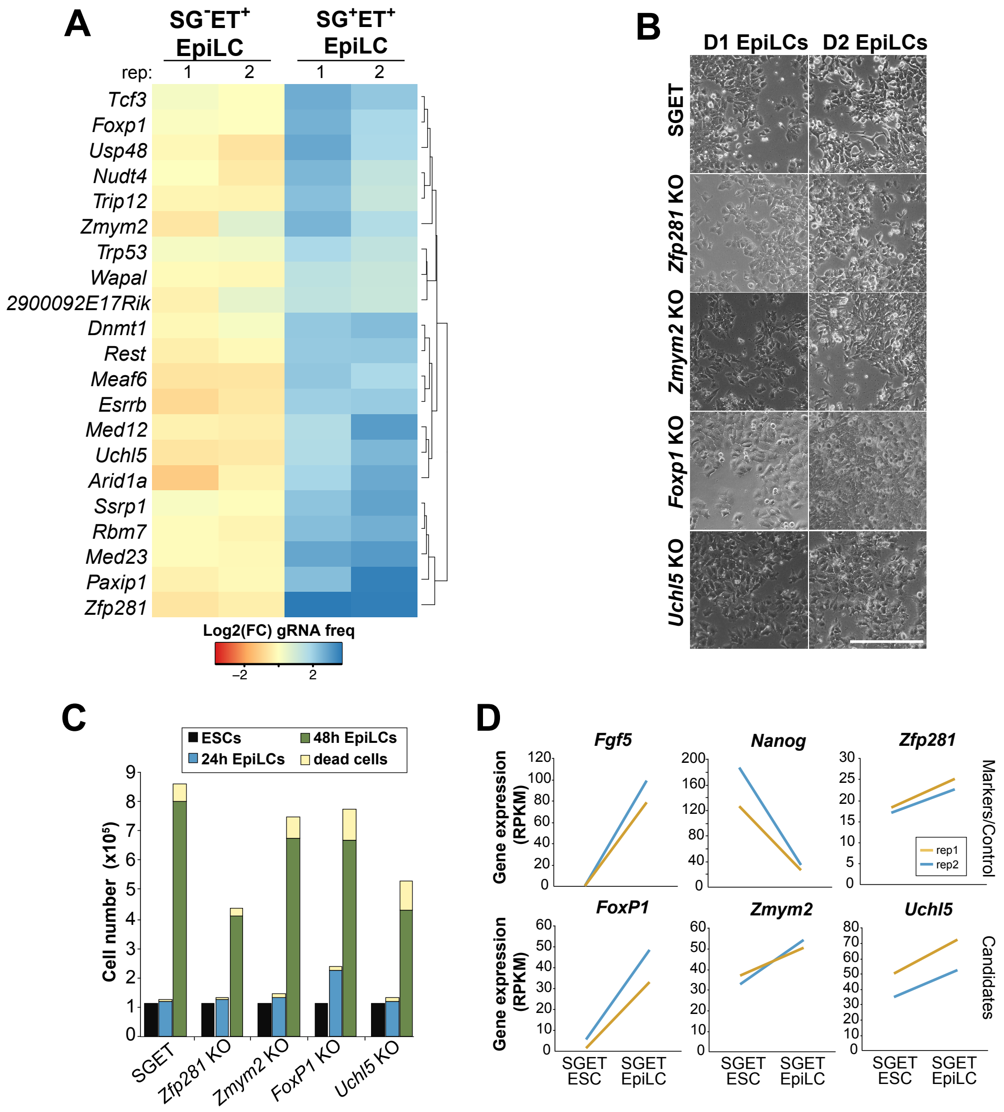

Supplementary Figure 3. CRISPR screen hits for candidate genes involved in exit from pluripotency. (A) Heatmap showing log2 fold-change (FC) of normalised gRNA frequency in SG-ET+ EpiLC that have appropriately exited naive pluripotency or SG+ET+ (*Stella*-positive) EpiLC that exhibit impaired exit, relative to normalised gRNA frequency in ESC. An enrichment of gRNAs in SG+ET+ EpiLC indicates that the targeted gene could be involved in dissolution of pluripotency. (B) Phase-contrast images of knockout EpiLC for candidate genes at day1 (d1) and day 2 (d2) of differentiation. (C) Number of cells after EpiLC induction of knockout candidate lines, indicating proliferation and survival. (D) RNA-seq data from WT SGET ESC and EpiLC shows the gene expression profile of markers (upper) and candidate genes for a role in exit from pluripotency after transition to EpiLC during the screen. Candidate genes are generally activated.

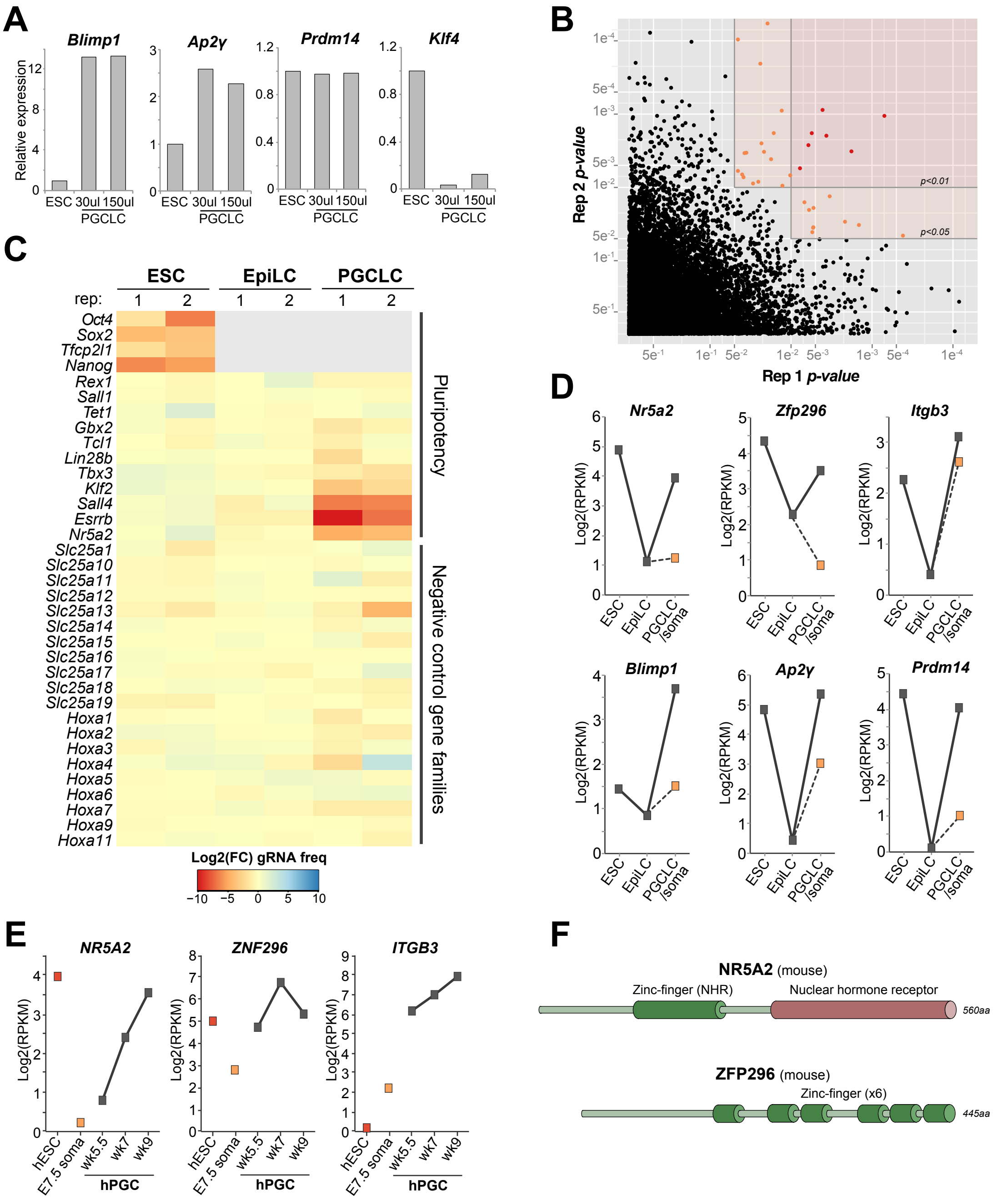

Supplementary Figure 4. CRISPR screen candidates for primordial germ cell (PGC) regulators. (A) Expression of key PGC genes in conditions used for the screen (30ul) and established conditions (150ul) by qRT-PCR. (B) Scatter plot showing significance scores of depleted genes across replicates. (C) Heatmap showing Log2 fold-change (FC) of normalised gRNA frequency (mean of up to 5 gRNA per gene) in each cell state relative to the preceding state (e.g. ESC is relative to initial library; EpiLC is relative to ESC; PGCLC is relative to EpILC). A depletion implies the gene targeted is important for that cell state. Shown are pluripotency genes and representative control gene clusters whereby knockout has no discernible effect of cell fate transitions. Note that knockout cells of some key naive pluripotency genes were highly depleted from the ESC population and are therefore uninformative for subsequent cell fates (shaded grey). Note *Sall4* is represented in the library by only a single gRNA. Of shown genes, only *Nr5a2* is statistically significant in PGCLC. (D) Gene expression profile of selected candidates and known PGC regulators in SGET ESC, SG-ET+ EpiLC and SG+ET- PGCLC during the screen, confirming the identity of each analysed population. Yellow indicates SG-ET- somatic cells. (E) Gene expression profile showing strong activation of PGC candidate regulators in developing human PGC in vivo. (F) Schematic showing protein structures of NR5A2 and ZFP296.

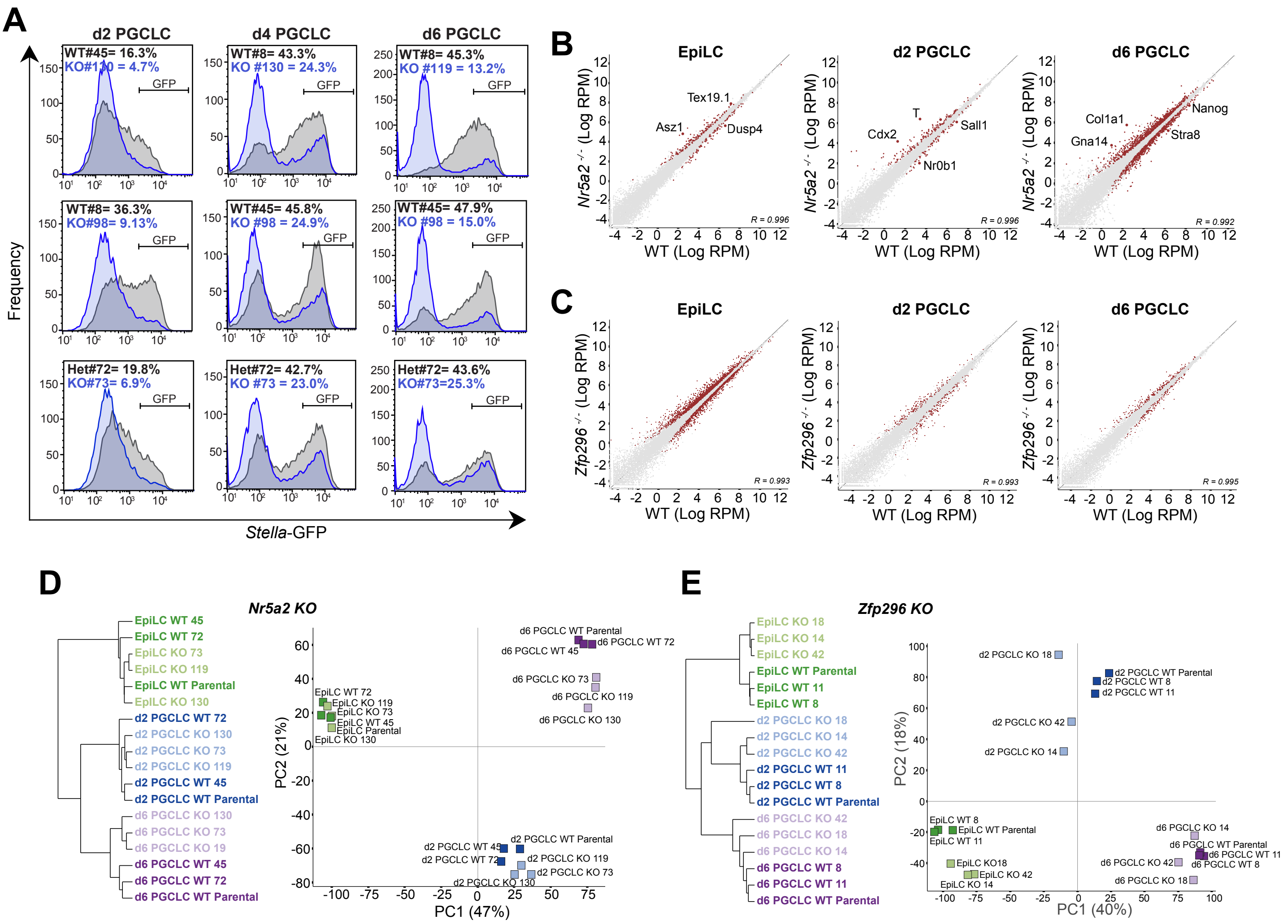

Supplementary Figure 5. Analysis of *Nr5a2* and *Zfp296* knockouts. (A) Representative FACS profiles from multiple independent *Nr5a2* KO lines during PGCLC induction. (B) Scatter plot showing expression of genes in *Nr5a2-/-* EpiLC and day 2(d2) and d6 PGCLC by RNA-seq. Significant ($p<0.05$) genes are highlighted red. (C) As above but for *Zfp296-/-* cells. (D & E). Unsupervised hierarchical clustering of RNA-seq data from *Nr5a2* knockout (KO) and matched WT controls (D), or *Zfp296* KO and controls (E), and 2D principal component analysis (PCA) using an alternative algorithm to Fig 4.

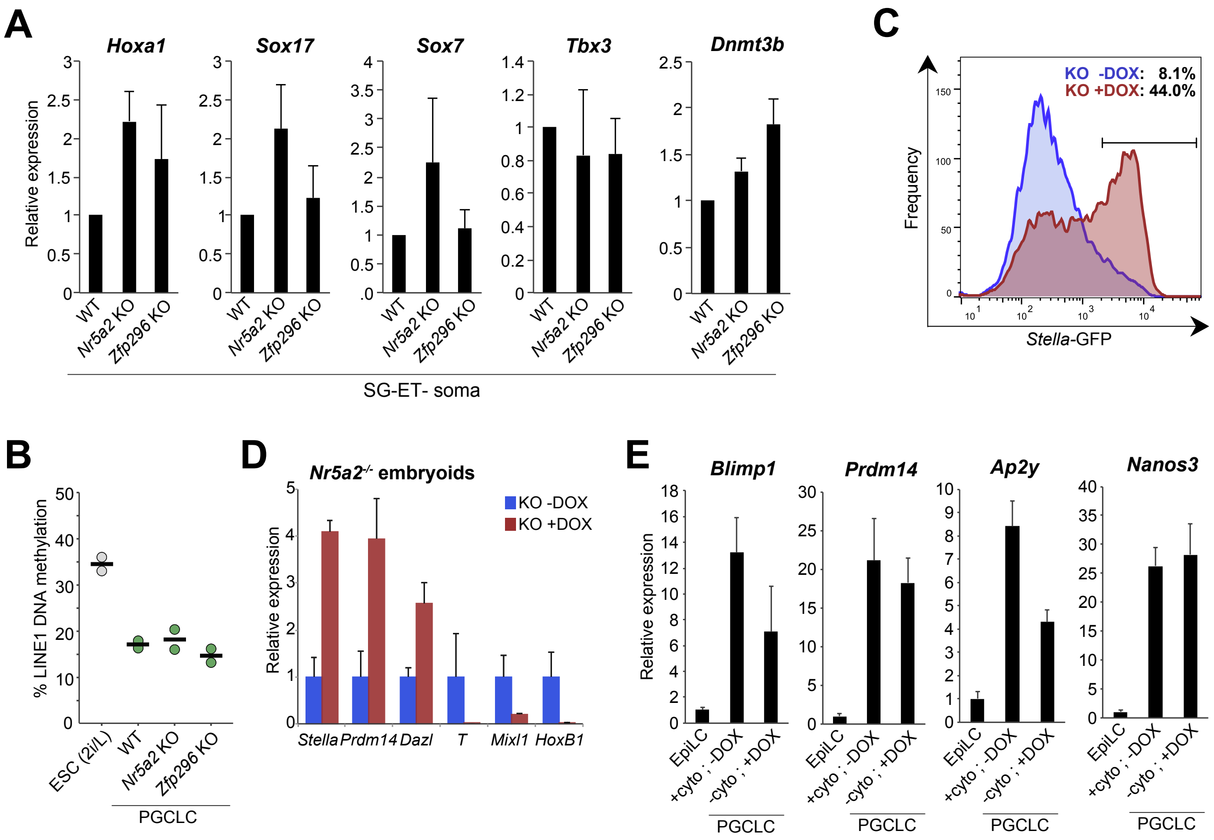

Supplementary Figure 6. Rescue of knockout PGCLC. (A) qRT-PCR of somatic cells that emerge during PGCLC differentiation at d6 showing expression of key somatic and lineage-specific genes. These SG-ET- soma cells were isolated during PGCLC induction (corresponding to 55-95% of cells), and imply that *Nr5a2* and *Zfp296* KO can apparently differentiate to somatic cell-types. (B) Percentage DNA methylation at LINE-1 elements in WT and mutant PGCLC as determined by bisulfite pyrosequencing, showing no difference in methylation erasure at these loci. (C) Additional representative FACS plot (related to Fig 6E) showing rescue of d2 PGCLC impairment in *Nr5a2* KO SGET lines by Dox-inducible expression of *Nr5a2* cDNA. Dox was added after EpiLC induction. (D) qRT-PCR expression in *Nr5a2* KO PGCLC-containing embryoids +/- Dox-induced activation of WT *Nr5a2*. Hyper-activation of mesodermal genes, such as *T*, is rescued by *Nr5a2* re-expression. (E) Expression of PGC regulators in control WT PGCLC (+Cyto; -Dox) or PGCLC derived by forced expression of *Nr5a2* without cytokines (-Cyto; +Dox) in WT PGCLC. Error bars in all panels represent s.e.m of duplicate biological experiments, with each experiment performed with at least 8 individual embryoids per sample.

Oligonucleotide sequences used in this study

qRT-PCR

**Target gene**	**Forward primer (5'-3')**	**Reverse primer (5'-3')**
Ap2y	ATCAAGATCGGACACCCAAC	ATGGCGATTAGAGCCTCCTT
Arbp (normaliser)	CAAAGCTGAAGCAAAGGAAGAG	AATTAAGCAGGCTGACTTGGTTG
Blimp1	GAGGATCTGACCCGAATCAA	CATGGAGGTCACATCGACAC
Dazl	CCAGAAGGCAAAATCATGCCAA	GGCAAAGAAACTCCTGATTTCGG
Dnmt3b	CTCGCAAGGTGTGGGCTTTTGTAAC	CTGGGCATCTGTCATCTTTGCACC
Fgf5	GGGATTGTAGGAATACGAGGAGTT	TGGCACTTGCATGGAGTTT
Gapdh (normaliser)	CCCCAACACTGAGCATCTCC	ATTATGGGGGTCTGGGATGG
Hey1	TCTGAGCTGAGAAGGCTGGTA	TCCACAGTCATCTGCAAGATCTCA
Hoxa1	GTGACTAGTCTTCTGCATGTCG	TCTGCTCTGGACCACATCACTC
Hoxb1	GATCCTACAGGTCTTGGGACC	AGCTCAAAGGCACTGAACTGAG
Klf4	GGGGTCTGATACTGGATGGA	CCCCCAAGCTCACTGATTTA
Mixl1	ACGCAGTGCTTTCCAAACC	CCCGCAAGTGGATGTCTGG
Nanog	CACCGTTCTCGGGATGAAAAACTGC	AAACGCAGTTTTTCATCCCGAGAAC
Nr5a2	ATGGGGAACAGGGGCAGATG	TCGCCACACACAGGACATAGC
Nr5a2	TGTGTGGCGATAAAGTGTCTG	TCGACAGTAGGGACATCGTTT
Nanos3	CACTACGGCCTAGGAGCTTGG	TGATCGCTGACAAGACTGTGG
Ogdh	ATGGAGGAGGAGGTGGCTATT	AGCCTTGGTTCTTGTGCTCTT
Otx2	CCACTTCGGGTATGGACTTG	GTCCTCTCCCTTCGCTGTTT
Pou2f3	AGAAACGGACCAGCATCGAG	AGATCTCTTCCGAGCTGGGTT
Prdm14	GCCTGAACAAGCACATGAGA	TGCACTTGAAGGGCTTCTCT
Prdm14	ACAGCCAAGCAATTTGCACTAC	TTACCTGGCATTTTCATTGCTC
Sox7	CCACAGTCCTTTGGCTGTCC	TACACGTGTCCAAGGGCAGA
Sox17	TTCTGTACACTTTAATGAGGCTGTTC	TTGTGGGAAGTGGGATCAAG
Stella	AGGCTCGAAGGAAATGAGTTTG	TCCTAATTCTTCCCGATTTTCG
T	CTGTGAGTCATAACGCCAGC	AGCCCCTTCATACATCGGAG
Tfcp2l1	CGGTGAAGCTACATGAAGAGACC	AGTCCCCTAGCTTCCGATTCT
Txn1	CATGCCGACCTTCCAGTTTTA	TTTCCTTGTTAGCACCGGAGA
Wnt3	CAAGCACAACAATGAAGCAGGC	TCGGGACTCACGGTGTTTCTC
Zfp296	CGTCAACTCCAAACTGCCTCG	AGATCTGGGTGCTTGTCGGTC
Zfp296	CGCGTAGATCCCGATACCG	GACGCTTCACATCCGATATGG

gRNA sequences and KO strategy

**Target gene**	**Target site**	**gRNA sequence (5'-3')**	**Strategy**
Zfp281	*Intron 1*	AACCCGTAAGTGTGGCCGGC	*Delete protein-coding potential by removing all the coding exons*
Zfp281	*3' UTR*	TCTGTACGTTATCACCGTAA	
Zmym2	*Exon 5*	CTGCTTTAACAGATACCGAA	*Generate frame-shifting indel(s) in coding exon*
FoxP1	*Exon 3*	TGACACTCGGTCCAACGGAG	*Generate frame-shifting indel(s) in coding exon*
Uchl5	*Exon 6*	TGAACTAGACGGGTTAAGAG	*Generate frame-shifting indel(s) in coding exon*
Zfp296	*Exon 1*	GTATCGGGATCTACGCGGCG	*Generate frame-shifting indel(s) in coding exon*
Nr5a2	*Intron 3*	CCCTATTAATTTCCCAATAT	*Generate frame-shifting deletion of critical exon for all isoforms.*
Nr5a2	*Intron 4*	GGAAGTGTCATAGAGCGACT	
Blimp1	*Intron 3*	CCGGCCAGCCCAAAGCGACC	*Generate frame-shifting deletion of critical exon for all isoforms.*
Blimp1	*Intron 4*	ACACATGACGAAGCCGTATA	

CRISPR library amplification including Illumina sequences

**Oligo**	**Sequence (5'-3')**
*Universal_F_*	AATGATACGGCGACCACCGAGATCTACACTCTTTCCCTACACGACGCTCTTCCGATCTCTTGTGGAAAGGACGAAACAC
Universal_F_+1stagger	AATGATACGGCGACCACCGAGATCTACACTCTTTCCCTACACGACGCTCTTCCGATCTTCTTGTGGAAAGGACGAAACAC
Universal_F_+2stagger	AATGATACGGCGACCACCGAGATCTACACTCTTTCCCTACACGACGCTCTTCCGATCTATCTTGTGGAAAGGACGAAACAC
Index2_R	CAAGCAGAAGACGGCATACGAGATACATCGGTGACTGGAGTTCAGACGTGTGCTCTTCCGATCCTAAAGCGCATGCTCCAGAC
Index5_R	CAAGCAGAAGACGGCATACGAGATCACTGTGTGACTGGAGTTCAGACGTGTGCTCTTCCGATCTAAAGCGCATGCTCCAGAC
Index6_R	CAAGCAGAAGACGGCATACGAGATATTGGCGTGACTGGAGTTCAGACGTGTGCTCTTCCGATCTAAAGCGCATGCTCCAGAC
Index12_R	CAAGCAGAAGACGGCATACGAGATTACAAGGTGACTGGAGTTCAGACGTGTGCTCTTCCGATCTAAAGCGCATGCTCCAGAC
Index19_R	CAAGCAGAAGACGGCATACGAGATCGTTTCACGTGACTGGAGTTCAGACGTGTGCTCTTCCGATCTAAAGCGCATGCTCCAGAC

gRNA adjacent binding site; Stagger; Index; Illumina TruSeq sequences

siRNA

**Target gene**	**Code**	**KD efficiency (mRNA) in ESC**
Ogdh	ON-TARGETplus siRNA *Ogdh* SmartPool (L-044219-01-0005)	76%
Txn1	ON-TARGETplus siRNA *Txn1* SmartPool (L-062799-01-0005)	84%
