# Supplementary material for: Tracing the transitions from pluripotency to germ cell fate with CRISPR screening
Source: Nat Commun. 2018 Oct 16;9:4292. doi: 10.1038/s41467-018-06230-0 (PMC6191455; doi:10.1038/s41467-018-06230-0)
Supplement: Supplementary file 3 — Description of Additional Supplementary Files [file 41467_2018_6230_MOESM3_ESM.pdf]

Description of Additional Supplementary Files

File Name: Supplementary Data 1

Description: Gene ontology (GO) terms and *p-values* for differentially expressed in KO lines during PGCLC induction and enriched populations in the CRISPR screen.
